# Variation in Eusperm Length May Reflect Reproductive Barriers and Differences in Sperm Competition Intensity Among *Littorina* Snails

**DOI:** 10.1002/ece3.71865

**Published:** 2025-08-03

**Authors:** Luisa Kumpitsch, Kerstin Johannesson, Jonathan N. Havenhand, Erica H. Leder

**Affiliations:** ^1^ Tjärnö Marine Laboratory, Department of Marine Sciences University of Gothenburg Strömstad Sweden; ^2^ Natural History Museum University of Oslo Oslo Norway

**Keywords:** ecotype, eusperm, reproductive barrier, sperm competition intensity

## Abstract

Reproductive barriers limit gene flow and drive population divergence. Sperm morphology plays an important role in reproductive barriers, as successful fertilization depends on how well sperm perform in the female environment. Specifically, sperm length must be adapted to fit the female reproductive tract and storage organs. We investigated sperm length in species and ecotypes of *Littorina*, a genus of promiscuous marine snails with internal fertilization. Various *Littorina* species have ecotypes adapted to different shoreline environments where reproductive traits like sperm length divergence might play a role in reinforcing these barriers. Due to their promiscuity, sperm competition likely plays a role in *Littorina*, and sperm length variation can give insights regarding sperm competition intensities. This study examined eusperm length in four species (*L. fabalis*, *L. littorea* , *L. obtusata* , *L. saxatilis* ) and two different ecotypes in both *L. fabalis* and *L. saxatilis* . The ecotypes of both *L. fabalis* and *L. saxatilis* differed in eusperm lengths, indicating the potential for this trait to be involved in prezygotic reproductive barriers between ecotypes of these species. Among‐species differences in eusperm length variation were observed and may be a result of different sperm competition intensities.

## Graphical abstract

Reproductive barriers, such as sperm morphology, play a key role in limiting gene flow and driving divergence in internally fertilizing species. We examined eusperm length variation in different *Littorina* species and ecotypes to assess its role in reproductive isolation and sperm competition. Eusperm length differed between ecotypes, suggesting its involvement in prezygotic barriers, whereas species and ecotype level differences may reflect varying sperm competition intensities.

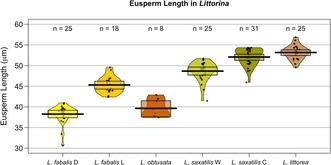

## 1 Introduction

Sperm cells are the most diverse cells in the animal kingdom, even though they fulfill the same function: the fertilization of an egg (Deneke and Pauli 2021; Lüpold and Pitnick 2018; Pitnick et al. 2009; Prakash et al. 2014). However, this diversity exists for a reason, as sperm must adapt to fulfill its function in a species‐specific manner yet move through a foreign environment before doing so. Sperm adaptation is especially relevant for internal fertilization, where sperm must pass through the female reproductive tract to find the fertilization site (Miller 2018; Suarez and Pacey 2006). Selection pressure within the female reproductive environment can lead to the rapid evolution of sperm traits (Fitzpatrick 2020), creating reproductive barriers between populations (Ahmed‐Braimah 2016; Eberhard 1996; Miller and Pitnick 2002; Pitnick et al. 2003). Reproductive barriers can reduce gene flow and promote divergence (Martin and Mendelson 2016). Sperm and female reproductive structures co‐evolve in many taxa, such as gastropods, passerine birds, and ground beetles (Beese et al. 2009; Briskie and Montgomery 1992; Sasakawa 2007), with considerable evidence showing that sperm length and the female storage organ can be closely linked (Minder et al. 2005). Sperm in passerine birds has also been described as a speciation phenotype, as selection to optimize performance in the female environment can reduce the variance in sperm length driving isolated populations apart and leading to post‐mating prezygotic isolation (Lifjeld et al. 2024).

Sperm competition is a form of postcopulatory sexual selection (Nixon et al. 2020; Parker and Pizzari 2010), and in its strictest definition, it refers to the competition of multiple males' ejaculates to fertilize a given set of ova (Birkhead and Immler 2007). With the transition to internal fertilization, sperm competition unfolds within the female reproductive tract, which sets the rules for competition and hence dictates the ejaculate features that enhance their competitiveness (Fitzpatrick and Lüpold 2014). Sperm competition drives the evolution of sperm traits and can be influenced by mechanisms related to female cryptic choice such as conspecific sperm capacitation (Parker 2020; Till‐Bottraud et al. 2005). Female cryptic choice can play a crucial role in post‐mating prezygotic isolation, as females may selectively store or utilize sperm based on male‐specific traits, thereby affecting fertilization success (Eberhard 1996; Firman et al. 2017; Poignet et al. 2022). Sperm competition intensity in externally and internally fertilizing species increases as more males compete for the same ova and can be quantified as the numbers of competing ejaculates (Engqvist and Reinhold 2007; Parker et al. 1996; Snook 2005). Rapid divergence in sperm length among populations of the same species reduces gene flow as sperm adapt to distinct female reproductive environments, establishing a post‐mating prezygotic barrier that promotes reproductive isolation (Kleven et al. 2008; Lifjeld et al. 2010, 2019, 2023, 2024).

Marine gastropods from the genus *Littorina* (Férrusac, 1822) are excellent model organisms for studying reproductive barriers and sperm competition. Some species evolve ecotypes, with morphological adaptations selected for different shoreline environments (Reid 1996). In *Littorina*, both females and males have been observed to mate with more than one partner, and several authors describe them as promiscuous (e.g., Erlandsson 2002; Erlandsson and Kostylev 1995; Panova et al. 2010). In *Littorina*, speciation is a multidimensional process (Johannesson et al. 2024; Westram et al. 2022), and there is still a lack of understanding of the factors that lead to divergence and speciation (Johannesson et al. 2024). One type of barrier that has hitherto not been considered in *Littorina* snails is post‐mating prezygotic isolation through sperm length divergence. *Littorina* snails have heteromorphic sperm, that is, two types of sperm: eusperm and (non‐fertilizing) parasperm (Buckland‐Nicks 1998). This study only focuses on the eusperm as it fertilizes the egg (Buckland‐Nicks et al. 1999; Buckland‐Nicks and Chia 1981). As it has been shown that sperm length divergence can act as a post‐mating prezygotic barrier by co‐evolving with specialized female storage organs (e.g., Cramer et al. 2021), we chose to measure eusperm cells, where length measurements are possible. In *L. saxatilis* , sperm can be stored and continue to fertilize eggs within the female for more than a year (Johannesson et al. 2016), which suggests that the eusperm have adapted to the female storage organ, as it must survive and be capable of fertilizing eggs within that time. During mating, female *Littorina* receive sperm through the gonopore. From there, it enters the *bursa copulatrix*, the first sperm storage organ. Later, the sperm is transferred and stored long‐term in the *receptaculum seminis* (Buckland‐Nicks and Chia 1990; Reid 1996). Fertilization occurs inside the “fertilization chamber,” a small area within the oviduct.

In this study we investigated eusperm length in *Littorina littorea* (Linnaeus, 1758), *Littorina obtusata* (Linnaeus, 1758) and the two distinct ecotypes of each of *Littorina fabalis* (Turton, 1825) and *Littorina saxatilis* (Olivi, 1792) on the Swedish west coast (Johannesson 2003; Johannesson et al. 2024; Le Moan et al. 2024; Reid 1996; Reid et al. 2012; Westram et al. 2021). *Littorina* snails copulate indiscriminately, leading to frequent interspecific copulations (Costa et al. 2020; Kemppainen et al. 2009; Maltseva et al. 2021; Mikhailova et al. 2009), highlighting the importance of post‐mating prezygotic mating barriers such as different eusperm lengths.

This research was conducted on the Swedish west coast. Here, *Littorina fabalis* exhibits higher genetic differentiation between its dwarf and large ecotypes (*F*_ST_ ~ 0.09) compared to the crab and wave ecotypes of *L. saxatilis* (*F*_ST_ ~ 0.04), largely due to inversions under divergent selection (Faria et al. 2019; Le Moan et al. 2023; Westram et al. 2021). This higher differentiation may suggest stronger reproductive barriers between *L. fabalis* ecotypes. In contrast, *L. saxatilis* shows weaker genetic isolation, consistent with more frequent hybridization in contact zones (Perini et al. 2020), which may be associated with less pronounced eusperm length divergence.

In *L. littorea* , which is distantly related to *L. saxatilis* , *L. fabalis*, and *L. obtusata* , genetic divergence between local populations is minimal (Janson 1987; Reid et al. 2012), suggesting weak reproductive isolation and potentially less selective pressure on eusperm length. Additionally, *L. fabalis* and *L. obtusata* overlap spatially and copulate (Hintz Saltin 2013), but viable hybrids are not found on the Swedish west coast (Kemppainen et al. 2009).

Although previous research has measured eusperm lengths in the species from the present study (Reid 1996), no study has specifically examined differences in eusperm length between ecotypes within *L. saxatilis* and *L. fabalis*. Given the documented genetic and phenotypic divergence between ecotypes (Johannesson et al. 2024; Le Moan et al. 2024; Westram et al. 2022), it is plausible that eusperm length varies between ecotypes, potentially acting as a reproductive barrier that reduces gene flow. We hypothesized that within *L. saxatilis* and *L. fabalis*, the different ecotypes would exhibit distinct total eusperm lengths, reflecting adaptation to the respective female reproductive environment. Additionally, we aim to investigate whether variations in eusperm length correlate with differences in sperm competition intensity. Specifically, we hypothesized that total eusperm length variance would be the lowest among males in *L. saxatilis* , as this species is known for high promiscuity (Panova et al. 2010) and, consequently, high sperm competition intensity. Lower eusperm length variance would indicate strong stabilizing selection on eusperm length in response to sperm competition intensity (Kleven et al. 2008; Lifjeld et al. 2019).

## 2 Materials and Methods

### 2.1 Sampling

All snails were collected in March 2023 and March 2024, which is within the reproductive period for all the sampled species (Johannesson et al. 2010). Snails were sampled from different islands in the Koster archipelago on the Swedish west coast. For each species/ecotype, only one population was sampled. On a rocky beach on Saltö, *L. saxatilis* crab ecotype and *L. littorea* were collected in the same 10 m^2^ area (geographic coordinates: 58°52′15.3″ N 11°07′11.9″ E), and *L. saxatilis* wave on a boulder cliff within a 2 m^2^ area (58°52′16.6″ N 11°07′04.1″ E). On Lökholmen, the *L. fabalis* large ecotype was sampled on a rocky beach, within a 10 m^2^ area (58°53′21.2″ N 11°06′32.3″ E), *L. fabalis* dwarf and *L. obtusata* in the same sandy bay, within a 20 m^2^ area (58°53′22.0″ N 11°06′38.1″ E). The sampling sites of *L. saxatilis* crab/ *L. littorea* and *L. saxatilis* wave were 250 m apart, and the sites of *L. fabalis* dwarf/ *L. obtusata* and *L. fabalis* large were 80 m apart from each other. The animals were brought to the Tjärnö Marine Laboratory facilities and kept at 4°C in seawater for a maximum of 48 h until further processing.

### 2.2 Sperm Measurement

To extract the sperm, seminal vesicles were dissected from the males and punctured with a needle to release the sperm. Approximately 50 μL of sperm were diluted in 200 μL PBS on a microscope slide. Sperm were imaged with a camera (Olympus OM‐D EM5ii) attached to a microscope (Olympus IX71) with a 10× objective. Images were uploaded to ImageJ (Schneider et al. 2012) for eusperm length measurements. A scale was applied to the images using an image of a Neubauer chamber (Assistent, Germany), and the total length of the eusperm cells was measured using the segmented line tool. Where possible, we measured ten morphologically normal cells per individual, but in some cases, it was impossible to find ten intact, elongated cells. Consequently, between 4–10 (6.44 ± 2.36, mean ± standard deviation) cells were measured for *L. fabalis* dwarf, 4–10 (8.06 ± 2.07) cells for *L. fabalis* large, 4–10 (8.28 ± 2.15) cells for *L. saxatilis* wave, 4–10 (8.44 ± 2.42) cells for *L. saxatilis* crab, 6–10 (7.38 ± 1.77) cells for *L. obtusata* , and 5–10 (9.52 ± 1.23) cells for *L. littorea* . Males with less than four intact elongated eusperm cells were discarded. The sample size *n* was 25 for *L. fabalis* dwarf, 18 for *L. fabalis* large, 8 for *L. obtusata* , 25 for *L. saxatilis* wave, 31 for *L. saxatilis* crab, and 25 for *L. littorea* . As sperm length may be correlated to body size (Gage 1994, 1998) we accounted for possible effects of body size by measuring the perimeter of the shell aperture (which is a good indicator of body size in *Littorina* snails and other gastropods, e.g., Ibom, Okon, and Bassey 2018; Kemp and Bertness 1984; Larsson et al. 2020; Phillips and Shima 2010; Smith 1981). Shells were photographed in a standardized way using a camera (Nikon D810) mounted on a stereo microscope (Olympus SZX16), and measurements were made in ImageJ (Schneider et al. 2012).

### 2.3 Statistical Analysis

All statistical analyses were performed in RStudio (Posit Team 2024; R version 4.4.1). All eusperm measurements from an individual were averaged. Finally, the mean, standard deviation (SD), and the coefficient of variance among males (CV_am_), that is, $\left(\frac{\mathrm{SD}}{\text{Mean}}\right)*100$, were calculated for each species/ecotype. To account for within‐male variance, the coefficient of variance within males (CV_wm_) was calculated using the eusperm length measurements of each individual male per species/ecotype. To reduce potential sources of (statistical) error, only one person was involved in measurements, and eusperm cells of five individuals per ecotype/species were measured in triplicate, chosen by a random sequence function call in RStudio (Posit Team 2024; R version 4.4.1). The repeatability of measurements was assessed with the R‐package *rptR* (Stoffel et al. 2017). Repeatability is estimated using a linear mixed model that includes a likelihood‐ratio test, testing whether repeatability is significantly different from zero (Stoffel et al. 2017). The repeatability measure represents the intra‐class correlation coefficient, that is, how much of the total variance in sperm length is explained by differences among males, where values closer to 1 indicate high repeatability. The repeatability *R* is calculated as $R=\frac{V_{G}}{V_{G}+V_{R}}$, where *V*_R_ is the within‐group (residual) variance and *V*_G_ + *V*_R_ “the fraction of the total phenotypic variance *V*_P_ in the population of interest that can be attributed to variation among groups *V*_G_” (Stoffel et al. 2017, p. 1639). The repeatability of measurements among the eusperm cells measured in triplicate of five males per species/ecotype was good (Table S3). All *p* values of the likelihood‐ratio test were smaller than 0.05 and *R*‐values close to 1 (Table S3), indicating high repeatability of the eusperm length measurements. To test for differences between the *L. fabalis* and *L. saxatilis* ecotypes, we applied linear mixed‐effects models using restricted maximum likelihood (REML) estimates with the response variable eusperm length, the fixed effect ecotype, and the random effect male ID using the R‐packages *lme4* and *lmerTest* (Bates et al. 2015; Kuznetsova et al. 2017). Model assumptions were evaluated by inspecting the residuals for normality and homogeneity of variance. Normality was assessed via quantile–quantile plots of the residuals, and homoscedasticity was checked by plotting residuals versus fitted values. Both, normality and homogenous variances of the model residuals were met. To test for a potential correlation between eusperm length and aperture perimeter in the studied *Littorina* species/ecotypes, we performed a Pearson's correlation test (Freedman et al. 2007) in R (Posit Team 2024; R version 4.4.1). Figures were created using the R‐packages *EnvStats* (Millard 2013), *ggplot2* (Wickham 2016), *dplyr* (Wickham et al. 2023) and *yarrr* (Phillips 2016) in RStudio (Posit Team 2024; R version 4.4.1) and the ImageJ (Schneider et al. 2012) plugin ScientiFig (Aigouy and Mirouse 2013).

## 3 Results

### 3.1 Eusperm Length Differences Among Ecotypes and Species

**FIGURE 1:**
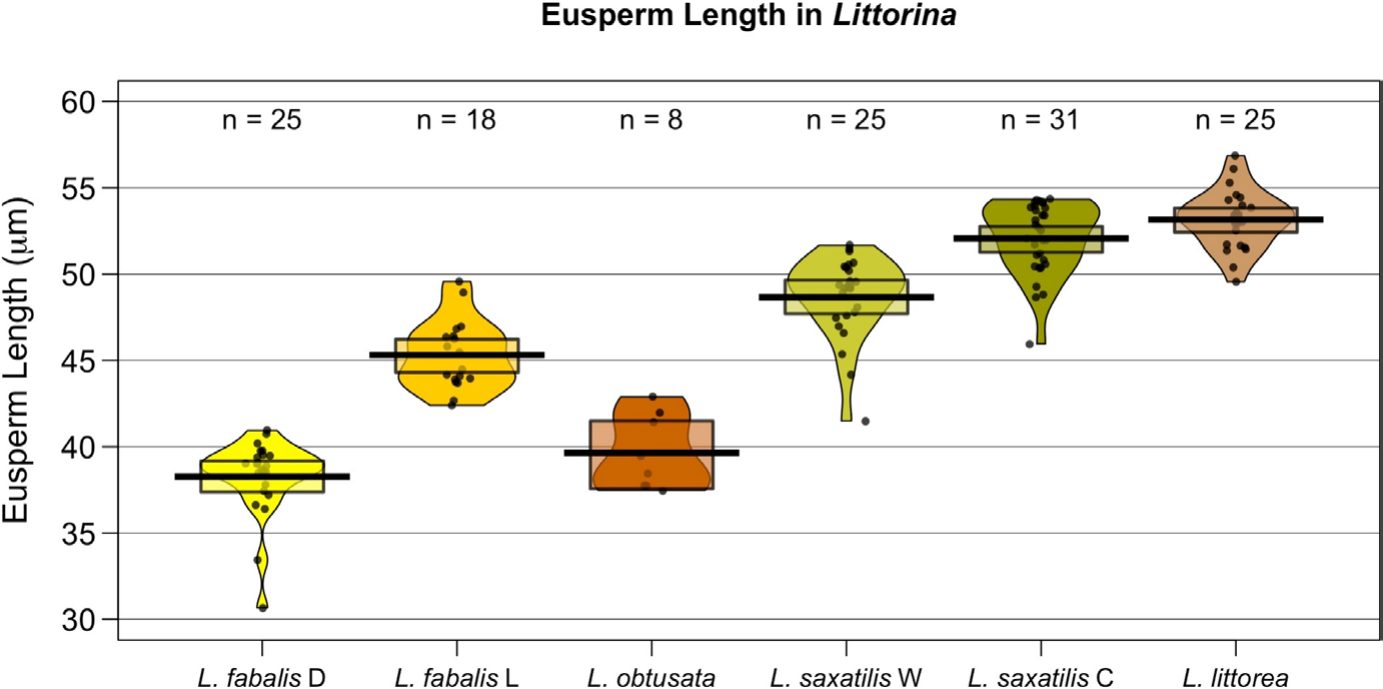
Eusperm length (μm) in *Littorina fabalis* ecotypes (D = dwarf; L = large), *L. obtusata* , *L. saxatilis* ecotypes (C = crab; W = wave) and *L. littorea* . NB: Black line within the box = mean, box = 95% confidence interval. The width of the density distribution reflects the frequency of observations at each eusperm length. Data points show the mean of each individual for which eusperm was measured.

Eusperm length significantly differed between the two *L. fabalis* ecotypes. The large‐ecotype males produced significantly longer eusperm (45.31 ± 1.99 μm, mean ± SD) than dwarf‐ecotype males (38.26 ± 2.22 μm; LME, *t* = 11.37, *p* < 0.0001; Table 1, Table S1, Figure 1). Similarly, in *L. saxatilis* , crab‐ecotype sperm (52.07 ± 2.07 μm) were longer than wave‐ecotype sperm (48.66 ± 2.39 μm; LME, *t* = −5.80, *p* < 0.0001; Table 1, Table S1, Figure 1). Among the other taxa, *L. obtusata* 's mean eusperm length (39.64 ± 2.16 μm) closely matched that of the *L. fabalis* dwarf ecotype (38.26 ± 2.22 μm), whereas *L. littorea* exhibited the longest eusperm of all species (53.16 ± 1.68 μm; Table 1, Figure 1).

**TABLE 1:** Mean eusperm length, standard deviation (SD), coefficient of variation among males (CV_am_), coefficient of variation within males (CV_wm_), population density and sires per brood of *Littorina fabalis* ecotypes, *L. saxatilis* ecotypes, *L. obtusata* , and *L. littorea* . For CV_am_, the mean of each male per species/ecotype was used.

Species	Ecotype	Mean eusperm length (μm)	SD (μm)	CV_am_	CV_wm_	Population density (individuals/m^2^)^a^	Sires per brood/mating period
*L. fabalis*	Dwarf	38.26	2.22	5.79	7.84	1.4	Not reported
	Large	45.31	1.99	4.40	7.92	1.4	Not reported
*L. saxatilis*	Wave	48.66	2.39	4.91	6.06	280	15–23^b^
	Crab	52.07	2.07	3.97	4.57	280	15–23^b^
*L. obtusata*	—	39.64	2.16	5.44	8.72	1.1	4–6^c^
*L. littorea*	—	53.16	1.68	3.16	5.41	2.3	Not reported

*Note:* For the CV_wm_, the eusperm measurements for each individual per species/ecotype were used. Data on population density refers to the Swedish west coast (see Johannesson et al. 2010). Sires per brood refer to the sires per mating period for *L. saxatilis* (Panova et al. 2010) and per brood for *L. obtusata* (Paterson et al. 2001).

^a^ Johannesson et al. (2010).

^b^ Panova et al. (2010).

^c^ Paterson et al. (2001).

### 3.2 No Strong Body‐Size Effect

**FIGURE 2:**
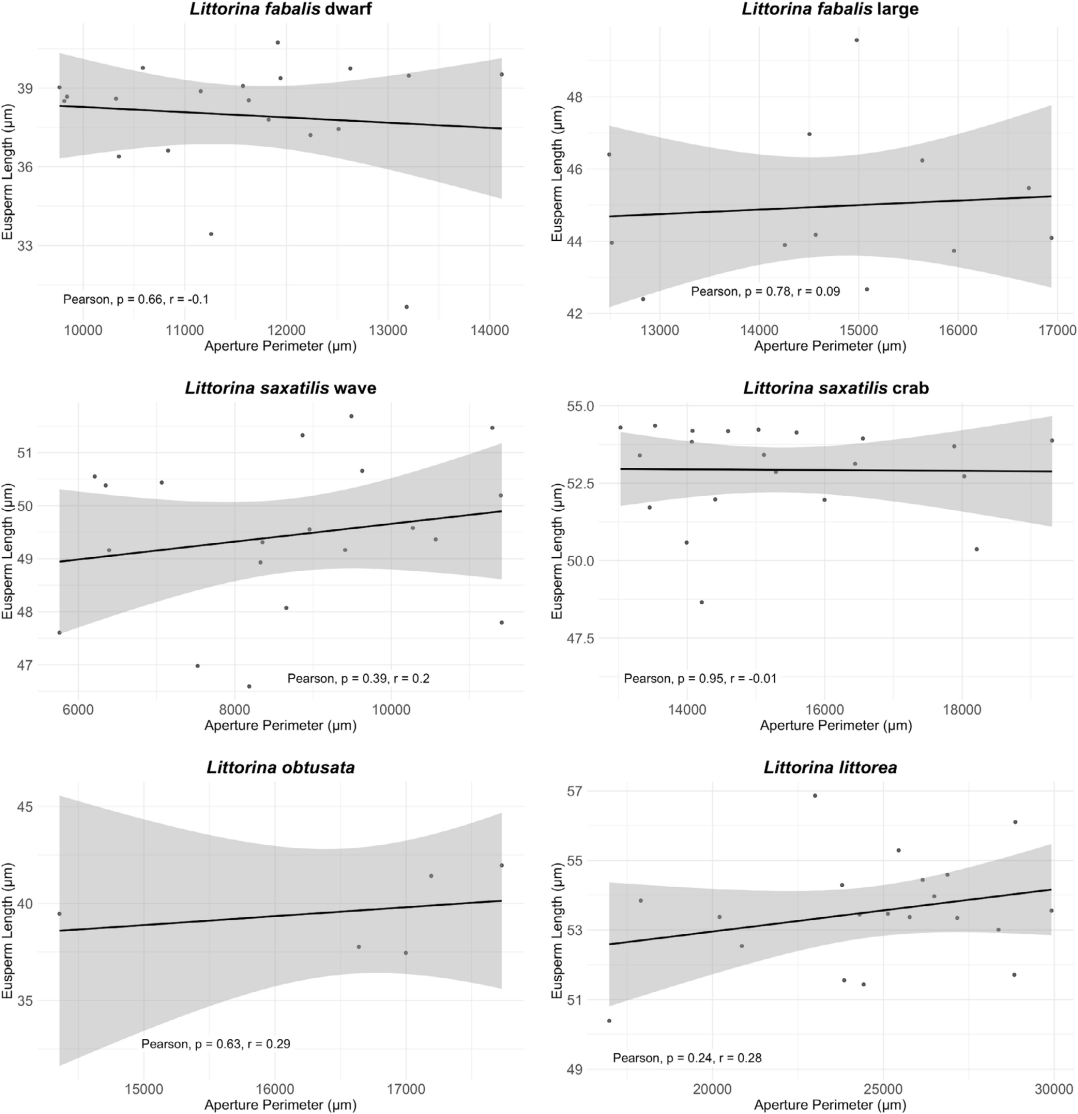
The scatterplots display the relationship between aperture perimeter (*x* axis) and eusperm length (*y* axis) for *Littorina fabalis* dwarf, *L. fabalis* large, *L. saxatilis* wave, *L. saxatilis* crab, *L. obtusata* , and *L. littorea* . Each data point represents an individual snail where eusperm length and aperture perimeter were measured. The black regression line indicates the trend in the data, with a shaded 95% confidence interval representing the uncertainty of the fit. A linear regression model quantifies this relationship. Pearson's correlation test (Freedman et al. 2007) showed no significant relationship between eusperm length and aperture perimeter for any of the species/ecotypes. The sample size was 20 individuals for *L. fabalis* D, 12 for *L. fabalis* L, 5 for *L. obtusata* , 20 for *L. saxatilis* W, 20 for *L. saxatilis* C and 20 for *L. littorea* . NB.: Pearson = Pearson's correlation test; *r* = correlation coefficient.

Across all six groups, eusperm length was not strongly associated with shell aperture perimeter (all *r* < 0.30, *p* > 0.05; Table S2; Figure 2), indicating that eusperm size differences were largely independent of overall body size.

### 3.3 Patterns of Variance

**FIGURE 3:**
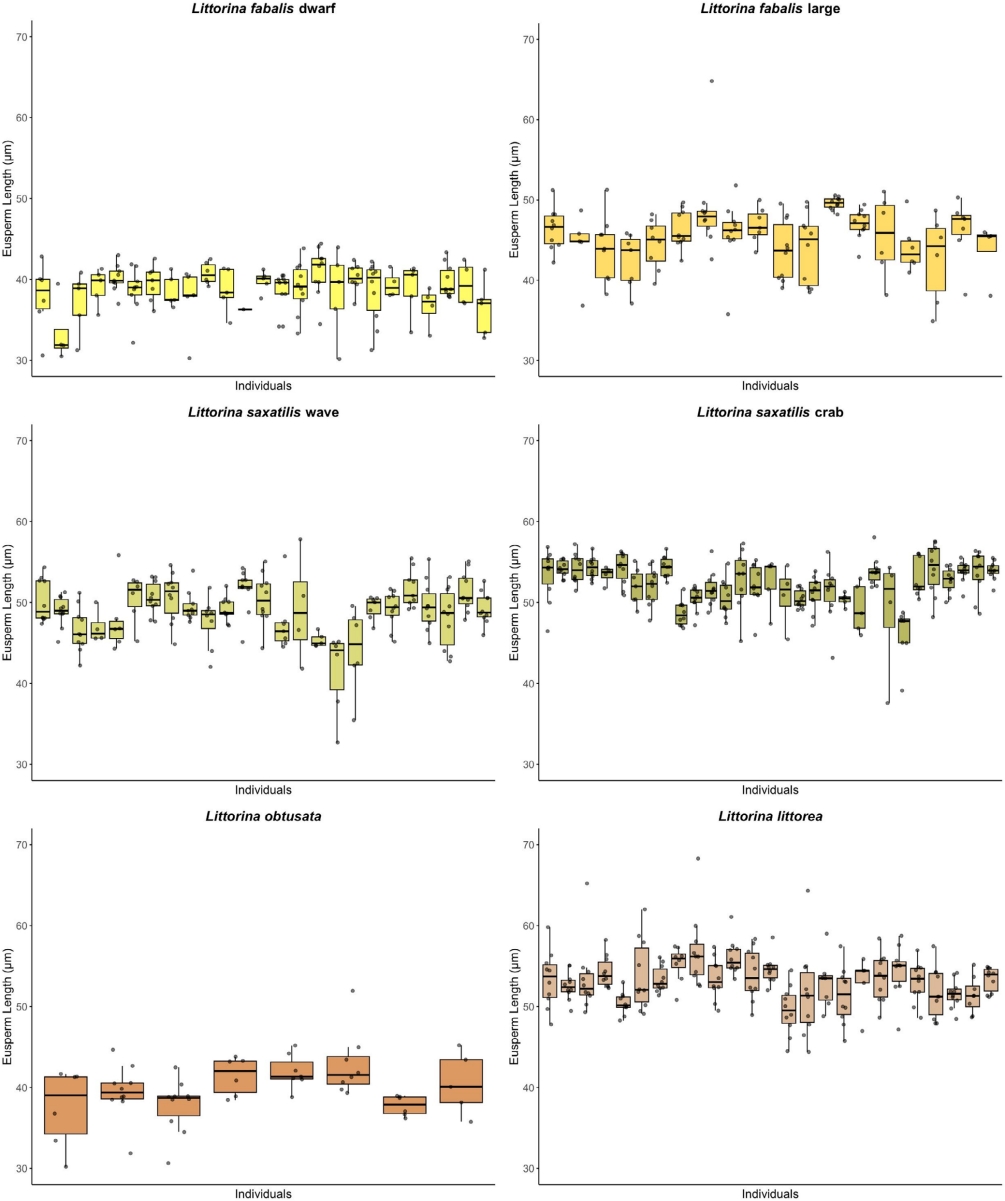
Within‐male eusperm length in *Littorina* species and ecotypes. Each boxplot represents one individual male, and each data point one eusperm cell within that male. NB: Black line = median, box = central 50% of the data. Vertical lines above and beneath the box show maximal and minimal data range. Data points out of that range are outliers.

When we partitioned within‐ versus among‐male variance in eusperm length, we found that among‐male variance was highest in the *L. fabalis* dwarf ecotype (CV_am_ = 5.79) and lowest in *L. littorea* (CV_am_ = 3.16; Table 1, Figure 3). Conversely, within‐male variance peaked in *L. obtusata* (CV_wm_ = 8.72) and was minimal in the *L. saxatilis* crab ecotype (CV_wm_ = 4.57; Table 1, Figure 3).

## 4 Discussion

### 4.1 Eusperm Length Differences Between *Littorina* Ecotypes Suggest a Post‐Mating Prezygotic Barrier

Our finding that mean eusperm lengths differed among *Littorina* ecotypes (within species), but were not correlated with body size, suggests that eusperm size may be a prezygotic reproductive barrier between these ecotypes. *L. fabalis* ecotypes meet in contact zones and create bimodal hybrid zones, in which parental genotypes are maintained, and only a few intermediates can be found (Le Moan et al. 2024). Hence, the ecotypes maintain their genetic and phenotypic integrity (Le Moan et al. 2024). The differences in eusperm length between *L. fabalis* ecotypes suggest this could play a role in maintaining this observed barrier to gene flow, generating genetic and phenotypic bimodality within hybrid zones (Johannesson et al. 2024; Le Moan et al. 2024). If the different eusperm lengths match differences in the female reproductive tracts between the ecotypes, this will further contribute to the reproductive barrier that limits hybridization. Furthermore, *Littorina fabalis* of the dwarf ecotype and the distinct species, *L. obtusata* , overlap on the shore and are observed to copulate (Hintz Saltin 2013), but there is no evidence of hybrids in the wild (Kemppainen et al. 2009). As the eusperm lengths of *L. fabalis* dwarf and *L. obtusata* are similar, it is unlikely that sperm length contributes to a post‐mating prezygotic barrier in this case. As these two species separated about 1 million years ago, multiple other barriers likely prevent hybridization (Reid et al. 2012; Hintz Saltin et al. 2013; Tatarenkov 1995) and hence selection on eusperm length might be weak. In a similar manner, differences in eusperm length in *L. saxatilis* might also play a role in post‐mating prezygotic reproductive isolation between ecotypes in the contact zone. Other prezygotic reproductive barriers in the study area (western Sweden) are relatively weak; for example, weak effects of assortative mating (Perini et al. 2020); low levels of genetic incompatibility between the 2 *L. saxatilis* ecotypes (Johannesson et al. 2024), and high levels of hybridization (Johannesson et al. 2024). Mean eusperm lengths were significantly different between ecotypes in this species; however, there was some overlap in length distributions (Figure 1), which suggests that any post‐mating prezygotic barrier established by eusperm length is probably not as strong as between *L. fabalis* ecotypes. Nonetheless, these results suggest that different eusperm lengths can be added to the list of possible mechanisms contributing to the multidimensional speciation processes of *L. fabalis* and *L. saxatilis* (see Johannesson et al. 2024).

### 4.2 Eusperm Length as an Adaptation to the Female Storage Organ

Sperm length has been shown to influence sperm capacity to reach and fit into the storage organ and fertilize the egg (Pitnick et al. 1999). Distinct differences in eusperm length suggest adaptation to different female reproductive environments. Although the dimensions of female sperm storage structures in *Littorina* remain unknown (and were not analyzed in this study), they are crucial for assessing eusperm adaptation. Sperm storage can be compartmentalized for short‐ and long‐term storage, like in the gastropod *Crepidula fornicate* (Beninger et al. 2016) or the spider *Dysdera erythrina* (Uhl 2000). In *Littorina*, these storage mechanisms also exist, by which eusperm from the *bursa copulatrix* (the short‐term storage organ) is transferred to the *receptaculum seminis* (the long‐term storage organ) where (as shown in *L. saxatilis* ) eusperm can remain viable for up to a year (Johannesson et al. 2016). This long‐term storage likely involves mechanisms to maintain sperm viability such as antioxidant production (Aitken 2020; Reinhardt and Ribou 2013; White et al. 2008) potentially facilitated by transferred parasperm proteins (Buckland‐Nicks and Fields 2020). There is speculation that proteins from the parasperm are transferred to the female (Buckland‐Nicks 1998; Lobov et al. 2015, 2018; Maltseva et al. 2022), but it has not been confirmed, and intact parasperm in *Littorina* (to our knowledge) have not been observed in the female reproductive tract. The observed eusperm length differences between ecotypes need to be matched with data on size differences of the *bursa copulatrix*, *receptaculum seminis*, and fertilization chamber in the females in order to more strongly support a hypothesis of prezygotic barriers and increase our understanding of sperm adaptation.

### 4.3 Eusperm Length Variation as an Indicator of Sperm Competition Intensity

High sperm competition intensity is associated with reduced variation in sperm length, reflecting strong stabilizing selection towards an optimal sperm length (Birkhead et al. 2005; Kleven et al. 2008). We are using the species' population density and, where it is available, sires per brood/mating period as already known proxies for sperm competition intensity here. *Littorina littorea* showed the lowest among‐male eusperm length variance, possibly reflecting high sperm competition intensity, supported by its relatively high population density (Table 1; Johannesson et al. 2010). The *L. saxatilis* crab ecotype had similarly low variance, consistent with its very high density (Table 1; Johannesson et al. 2010) and many sires per brood (Table 1; Panova et al. 2010). In contrast, eusperm length in the wave ecotype showed higher variance, though reported densities and paternity rates are the same between ecotypes, suggesting additional factors may play a role (Janson 1982; Johannesson et al. 2010; Makinen et al. 2007; Panova et al. 2010). *L. fabalis* large ecotype showed lower variance than the dwarf ecotype, which could imply higher sperm competition; however, field observations suggest lower densities in the large ecotype, questioning this interpretation. In addition to sperm competition, other factors, such as resource availability, abiotic variables, seasonality, genetic constraints, etc., can influence sperm length variances (Blanckenhorn and Hellriegel 2002; Dufour et al. 1984; Evans 2011; Macartney et al. 2019). *L. obtusata* , with the lowest density (Table 1; Johannesson et al. 2010), had the highest among‐male variance, suggesting low sperm competition. Although paternity data for *L. obtusata* show multiple sires (Table 1; Paterson et al. 2001), the number remains lower than in *L. saxatilis* . Across all taxa, within‐male variance exceeded among‐male variance, possibly due to variability during spermatogenesis or adaptive flexibility in eusperm length, which is especially relevant in taxa where females store sperm (Reinhardt et al. 2015) and female cryptic choice plays a role—for example, in bumblebees, where sperm length variation decreases after female storage (Baer et al. 2003). Among‐male eusperm length variance is shaped more by competitive pressures, as males with suboptimal eusperm lengths are less likely to succeed (Kleven et al. 2008). Together, these patterns suggest that while stabilizing selection reduces among‐male variance under high sperm competition intensity, within‐male variance may persist due to both developmental constraints and potential adaptive roles to female storage organ variability.

## 5 Conclusion

Our study revealed that eusperm length differs significantly between *Littorina* ecotypes of the same species, suggesting potential adaptation to distinct female reproductive environments. Although these differences do not comprise direct evidence of a post‐mating prezygotic barrier, they indicate that eusperm length may contribute to reproductive isolation, in combination with other factors. Additionally, eusperm length variation among males differed across species, potentially reflecting differences in sperm competition intensity. Yet, evidence for this link is limited, and alternative influences—such as female‐mediated selection and sperm storage—may play significant, poorly understood roles. As only one population per species and ecotype was sampled, we cannot extrapolate our findings to the whole distributions of these species, indicating the need to investigate more populations in future studies. Further research into the female reproductive tract and the role of parasperm is essential to better understand how eusperm length variation contributes to reproductive isolation and competition among *Littorina* species.

## Author Contributions

**Luisa Kumpitsch:** conceptualization (equal), data curation (lead), formal analysis (lead), funding acquisition (equal), investigation (lead), methodology (equal), writing – original draft (lead), writing – review and editing (equal). **Kerstin Johannesson:** conceptualization (equal), formal analysis (supporting), funding acquisition (equal), investigation (supporting), methodology (equal), resources (equal), writing – original draft (supporting), writing – review and editing (equal). **Jonathan N. Havenhand:** conceptualization (equal), formal analysis (supporting), funding acquisition (equal), investigation (supporting), methodology (equal), project administration (supporting), resources (equal), supervision (supporting), writing – original draft (supporting), writing – review and editing (equal). **Erica H. Leder:** conceptualization (equal), formal analysis (supporting), funding acquisition (equal), investigation (supporting), methodology (equal), project administration (lead), resources (equal), supervision (lead), writing – original draft (supporting), writing – review and editing (equal).

## Conflicts of Interest

The authors declare no conflicts of interest.

## Supporting information


**Table S1.** ece371865‐sup‐0001‐TablesS1‐S3.docx.
**Table S2.** ece371865‐sup‐0001‐TablesS1‐S3.docx.
**Table S3.** ece371865‐sup‐0001‐TablesS1‐S3.docx.

## Data Availability

Data are available from the SciLifeLab Data Repository, https://doi.org/10.17044/scilifelab.28590188.v2.
