# Supplementary material for: Variation in Eusperm Length May Reflect Reproductive Barriers and Differences in Sperm Competition Intensity Among Littorina Snails
Source: Ecol Evol. 2025 Aug 3;15(8):e71865. doi: 10.1002/ece3.71865 (PMC12318614; doi:10.1002/ece3.71865)
Supplement: Supplementary file 1 — Table S1. ece371865‐sup‐0001‐TablesS1‐S3.docx. Table S2. ece371865‐sup‐0001‐TablesS1‐S3.docx. Table S3. ece371865‐sup‐0001‐TablesS1‐S3.docx. [file ECE3-15-e71865-s001.docx]

**Supporting Information**

*Table S1: Results of the linear mixed-effects models using restricted maximum likelihood estimates with the fixed effect ecotype and the random effect male ID. Models were conducted to test for differences in the response variable eusperm length between ecotypes in the species Littorina fabalis and Littorina saxatilis. Estimate, standard error (SE), degrees of freedom (df), t-value and p-value are shown. The results were obtained by applying the package lme4 and lmerTest (Bates et al., 2015; Kuznetsova et al., 2017) in RStudio (Posit Team, 2024; R version 4.4.1).*

| **Species** | **Estimate** | **SE** | **df** | **t-value** | **p-value** |
| --- | --- | --- | --- | --- | --- |
| *L. fabalis* | 7.00 | 0.62 | 36.31 | 11.37 | < 0.0001 |
| *L. saxatilis* | -3.39 | 0.58 | 51.59 | -5.80 | < 0.0001 |

*Table S2: Pearson’s correlation test results of the correlation between eusperm length and aperture perimeter in Littorina species and ecotypes. The results were obtained by applying the Pearson’s correlation test in RStudio (Posit Team, 2024; R version* *4.4.1). NB: df= degrees of freedom, r= correlation coefficient.*

| **Species** | **Ecotype** | **t-value** | **df** | **p-value** | **r** |
| --- | --- | --- | --- | --- | --- |
| *L. fabalis* | dwarf | -0.44 | 18 | 0.66 | -0.1 |
| *L. fabalis* | large | 0.29 | 10 | 0.78 | 0.09 |
| *L. saxatilis* | wave | 0.88 | 18 | 0.39 | 0.2 |
| *L. saxatilis* | crab | -0.06 | 19 | 0.95 | -0.01 |
| *L. obtusata* | / | 0.53 | 3 | 0.63 | 0.29 |
| *L. littorea* | / | 1.22 | 18 | 0.24 | 0.28 |

*Table S3: Repeatability (R), Standard Error (SE), 95% Confidence Interval (CI) of the repeatability and p-value of the Likelihood-Ratio-Test (LRT) in Littorina species and ecotypes. The results were obtained by using the R-package rptR (Stoffel et al., 2017) in RStudio (Posit Team, 2024; R version* *4.4.1). NB: repeatability measure represents the intra-class correlation coefficient, i.e.* *how much of the* *total* *variance in sperm length is explained by differences among species/ecotypes and not due to measurement error.*

| **Species** | **Ecotype** | **R** | **SE** | **95% CI of R** | **p-value of LRT** |
| --- | --- | --- | --- | --- | --- |
| *L. fabalis* | dwarf | 0.87 | 0.04 | 0.77 - 0.92 | p < 0.0001 |
| *L. fabalis* | large | 0.97 | 0.009 | 0.95 - 0.98 | p < 0.0001 |
| *L. saxatilis* | wave | 0.92 | 0.02 | 0.87 - 0.95 | p < 0.0001 |
| *L. saxatilis* | crab | 0.93 | 0.02 | 0.89 - 0.96 | p < 0.0001 |
| *L. obtusata* | / | 0.95 | 0.02 | 0.91 - 0.97 | p < 0.0001 |
| *L. littorea* | / | 0.96 | 0.01 | 0.93 - 0.97 | p < 0.0001 |
